# Exploring the metabolic and antioxidant potential of solergy: Implications for enhanced animal production

**DOI:** 10.1016/j.btre.2023.e00821

**Published:** 2023-11-28

**Authors:** Pamela Olivares-Ferretti, Viviana Chavez, Ekaitz Maguregui, Silvia Jiménez, Octavi Colom, Jorge Parodi

**Affiliations:** a Laboratorio de Investigación Biosocial, Tonalli ltda, Temuco, Chile; b Igusol Advance S.A., Navarrete, Spain

**Keywords:** Cells, Animal production, Antioxidante, Glcogen, Metabolism

## Abstract

Cell models are indispensable tools in biotechnology when investigating the functional properties of organic compounds. The emergence of various additives designed to enhance animal production has introduced the need for in-depth evaluations, which are often hindered by the complexities of *in vivo* testing. In this study, we harnessed cell-based models to scrutinize the impact of Solergy as a regulator of cellular metabolism with a particular focus on its modulation of glycogen and antioxidant effects. Our experiment was designed to include assessments of the influence of Solergy on the viability of both terrestrial and aquatic vertebrate cell models, which revealed the benign nature of Solergy and its lack of adverse effects. Furthermore, we examined the capacity of Solergy to modulate intracellular ATP concentrations and enhance glycogen accumulation. Notably, the antioxidant potential of Solergy and its ability to mitigate cellular aging were evaluated within the same cellular frameworks. The outcomes of our investigation suggest that Solergy is a potent metabolic regulator that elevates cellular activity while exerting an antioxidant effect. Importantly, our study demonstrates that Solergy does not induce changes in membrane oxidation. These findings indicate the potential of using Solergy to regulate glycogen synthesis, intracellular ATP concentrations, and oxidative stress in production animals. The multifaceted effects of this additive, which acts as both a metabolism enhancer and an antioxidant, open doors to the creation of custom diets tailored to meet specific production needs while maintaining stable production parameters.

## Highlights

• The nutrition of animal production needs continuous control, and new additives need to be tested.
• Antioxidants are essential for the development of new diets in animal production.
• Cell research helps to reduce the cost and time of exploring this new molecule, especially antioxidants.
• Our results present new evidence for explaining the action mechanism for new molecules at the cell level and can used for future applications in animal nutrition.

## 1 Introduction

Cell models are widely employed as screening systems for innovative products. Such models are frequently utilized to evaluate novel molecules, vaccines, and cellular mechanisms [1,2], and various models are used to understand different mechanisms; for example, mouse cells are used to study the effects of ethanol and to understand Alzheimer's disease [3].

The observation of cell development as an indicator of production remains a novelty. Data obtained from studies involving salmonid cells have made it possible to regulate the dosage of additives in diets using compounds such as nucleotides to enhance development under fasting conditions in production facilities [4]. Another study observed synergistic effects between two different molecules used as antioxidants in diets, which improves the concentration of each in the salmon diet [5]. Without these investigations, it would take longer to develop diets that can impact production.

Growth stimulation models need energy, and the regulation of ATP concentrations relies heavily on glucose metabolism. Regulating ATP and glycogen levels in cells is pivotal to understand energy reserves [6]. However, such modulation results in the generation of waste, such as free radicals, and oxidative stress, which indicates that the molecules that promote growth generate ATP and are metabolically active [7,8].

Administering energy-rich diets to fish has been reported to have adverse effects, such as the generation of resistance to lipid transport, inflammation, and pathologies such as cataracts [9,10]. Therefore, reducing the effects of metabolic waste generated from high-energy diets by using additives can improve production performance [11].

Additionally, these models have been employed for comprehension; for instance, to understand the mechanism of reducing oxidation at the membrane level and cellular responses [12,13]. An antioxidant agent is a compound that aids in the restoration of the balance of reactive oxygen species [14,15]. Reactive oxygen species form during normal cell metabolism and fulfil a physiological function as a signal of cellular catabolism [16].

However, under certain conditions, such as an excess of nutrients or the induction of alternative biochemical pathways, more molecules are generated and the protection mechanisms at the cellular level are overwhelmed, which creates a toxic environment for cells and consequently for the organism [17,18]. In this context, incorporating compounds, additives or molecules to reduce the concentration of free radicals and generate a better cellular response [19,20] would promote the health and growth of production animals [21].

The objective of this study was to demonstrate that the compound Solergy is an intriguing additive by assessing both its capacity to provide energy at the cellular level as well as its antioxidant capability, and the results showed that Solergy synergistically complements the metabolic enhancement and thereby reduces the impact of free radicals. Moreover, the concentrations of intracellular ATP, as an energy source, were measured, and specifically, the ability of Solergy to increase the glycogen reserve and thus achieve improved production performance was determined. The results obtained at the cellular level indicate that Solergy can metabolically stimulate growth and reduce oxidative stress.

## 2 Methodology

### 2.1 Molecule

Solergy is an organic additive registered under the trademark Solergy® that is manufactured by Igusol Advance SA (Navarrete, Spain). This water-soluble biodegradable compound is registered as a nonhazardous substance.

### 2.2 Cell culture and determination of LD50

This study utilized CHSE-214 (*Oncorhynchus tshawytscha* embryo) and LMH (*Gallus gallus* hepatocytes) cell cultures. These cell models were chosen due to their relevance in commercial production, particularly within the context of the application of Solergy as an energy supplement for poultry (LMH cells) and aquaculture industries (CHSE-214 cells). Our experimental methodology, cell culture media, and handling procedures were adapted from those described by Olivares et al*.* [4]. Each graph represents the results from three independent experiments, and each of these experiments was conducted with three replicates. Both cell lines were exposed to different concentrations of the additive ranging from 0 to 200 g/L for varying durations to assess its toxicity and determine its cellular safety threshold, which is represented by the LD50.

### 2.3 ATP measurements

To evaluate the potential of using Solergy as an energy utilization enhancer in food, we analyzed several factors. We quantified the intracellular energy levels by measuring the ATP-ADP levels through luminometry using MaxL spectral luminometry equipment. LMH cells were subcultured and subjected to treatment under different glucose and Solergy conditions. Solergy concentrations in the range from 0 to 20 g/L were used for the ATP and ATP/ADP ratio measurements, whereas the cell viability relative to ATP concentration was assessed using Solergy concentrations in the range of 0.001 to 10% g/L. The glucose concentrations used ranged from 0 to 50 mM. The samples were compared with a reagent blank, and the concentrations were estimated using an ATP calibration curve with units of nanomolar (nM).

### 2.4 Glycogen assessment

In line with the potential offered by Solergy, LMH cells were subcultured and exposed to glucose (25 g/L) and Solergy at concentrations of 0.2 and 2 g/L for 12 h. Subsequently, two additional experiments to quantify glycogen content were conducted using Solergy concentrations ranging from 0 to 20 g/L and glucose concentrations ranging from 0 to 25 mM for 24 h. Finally, a time curve was established after incubation of LMH cells with 25 mM glucose and 2 g/L Solergy. To determine the intracellular glycogen levels, a PAS rapid staining kit was employed. The stained cells were meticulously examined under an inverted Nikon phase-contrast microscope and documented using a C-Mos digital camera. ImageJ software was harnessed for image analysis, which enabled the quantification of glycogen concentrations.

### 2.5 Pharmacological antioxidant assessments

In addition to its primary function, the potential antioxidant properties of the Solergy molecule were also examined. To evaluate these properties, pharmacological antioxidant tests were conducted using two distinct approaches.

First, we employed a fixed hydrogen peroxide (H_2_O_2_) concentration of 100 µM in combination with varying concentrations of the compound (from 0 to 20 g/L) with both cell lines. This approach allowed the concentration at which the additive shielded cells from oxidative damage to be determined.

Second, we employed a pharmacological strategy previously utilized for assessing antioxidant capacity [5,22]. In this method, H_2_O_2_ was used at concentrations ranging from 0.1 µM to 1000 µM to directly induce oxidative stress, and the results were compared with Solergy concentrations of 0.2 and 2 g/L. This specific concentration was validated using a previously established safety curve.

### 2.6 Cell aging evaluation

Furthermore, to evaluate cell aging, the Abcam TBARS kit was used. The TBA reagent (200 µL) was added to both standards and samples. Subsequently, the absorbance of each sample was measured at an OD of 535 nm (525 to 545 nm) to evaluate color formation. This OD value is proportional to the concentration of oxidized lipids, from which the oxidation state in the cells can be determined. The same cytotoxicity curves were employed to correlate mortality with aging.

### 2.7 Data analysis

The results from the image analyses are presented as the averages ± SEMs. Statistical comparisons were conducted using Student's t-test or ANOVA. A probability level (p value) lower than 0.05 was considered to indicate statistical significance. All statistical analyses were performed using GraphPad PRISM 9.0 software (GraphPad Software, La Jolla, California, U.S.A.).

## 3 Results

### 3.1 Cellular safety

**Fig. 1:**
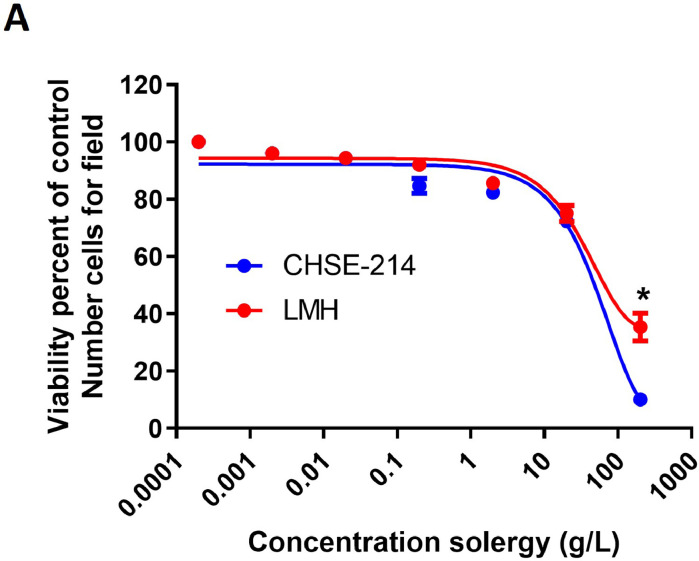
**Safety curve**. Curves of the viability of CHSE-214 and LMH cells in response to increasing concentrations of Solergy. The data points represent the means ± SEMs.

To determine the activity of Solergy and its recommended dose, a safety curve of the additive was developed. Both CHSE-214 (trout embryo) and LMH (rooster hepatocyte) cells were exposed to increasing concentrations of the compound for 24 h. The cell counts per unit area were determined, and cell viability was estimated. As shown in Fig. 1, the LD50 of Solergy was found to equal 20 g/L, indicating that the recommended doses fall below the lethality threshold. Solergy concentrations exceeding 200 g/L reduced cell viability, demonstrating the biological activity of the compound.

### 3.2 Measurement of the intracellular ATP level

**Fig. 2:**
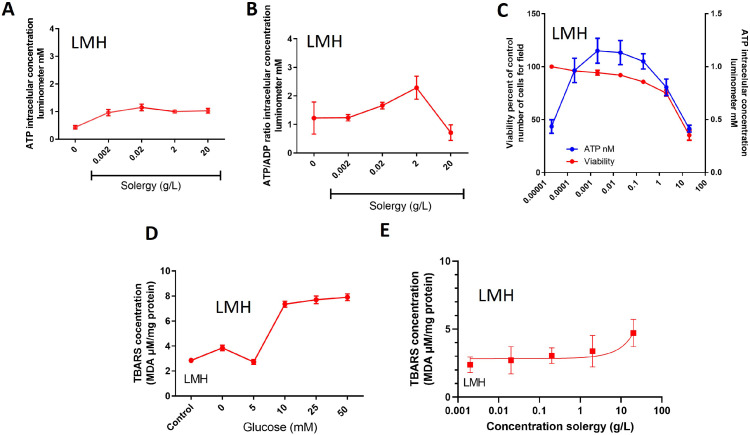
**Energetic effects of Solergy**. (A) Intracellular ATP concentration in LMH cells incubated with increasing Solergy concentrations. (B) Intracellular ATP/ADP ratio in LMH cells incubated with increasing concentrations of Solergy. (C) Relationship between cell viability and ATP concentration in LMH cells incubated with increasing Solergy concentrations. Furthermore, the TBARS values for LMH cells exposed to increasing concentrations of (D) glucose and (E) Solergy were determined. The points represent the means ± SEMs.

The concentration of ATP and the ATP/ADP ratio in LMH cells provide insights into the cellular metabolic response to a stimulus. Extreme ATP concentrations, whether low or high, indicate metabolic stress. Additionally, a reduced ATP/ADP ratio suggests high energy consumption and also reflects cellular stress. In this context, Solergy appears to elevate the ATP concentration while maintaining an ATP/ADP ratio close to the baseline conditions, implying that this additive contributes to energy preservation without inducing significant metabolic stress (Fig. 2A and B). Cell viability was found to be positively correlated with the ATP concentration in LMH cells, as shown in Fig. 2C. These results suggest that Solergy enhances cell metabolic activity without compromising viability, potentially due to its capacity to mitigate oxidative stress.

In contrast, glucose exposure resulted in an increase in the ATP concentration with a concurrent decrease in cell viability and possibly heightened oxidative stress, as demonstrated in Fig. 2D. To assess this effect, we examined LMH cell membrane oxidation after exposure to glucose and Solergy. As illustrated in Fig. 2D, glucose-exposed cells experienced more pronounced oxidation, whereas the addition of Solergy did not induce the same increase, as shown in Fig. 2E. These observations hint that Solergy protects against oxidation, which is observable as a secondary effect of increased metabolic activity due to heightened energy levels. The antioxidant properties of Solergy counteract the potential detrimental impact of its promotion of high metabolic activity without causing adverse effects, which consequently bolsters cell viability even under metabolic stress.

### 3.3 Measurement of intracellular glycogen levels

**Fig. 3:**
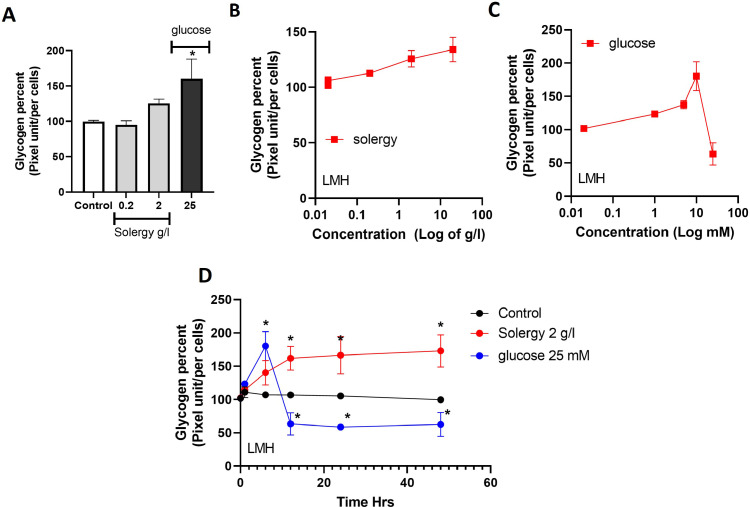
**Effects on the intracellular glycogen concentration**. Glycogen levels in treated LMH cells. (A) Glycogen concentration in cells exposed to the indicated conditions. (B) Glycogen level after 24 h of incubation with increasing concentrations of Solergy. (C) Graph of the glycogen levels in cells exposed to Solergy. (D) Time curve of the glycogen level during incubation under the indicated conditions. The points and bars indicate the averages ± SEMs.

Glucose has a significant impact the regulation of cellular energy and can trigger cell death due to oxidative stress. Thus, mitigating this undesired effect and promoting glycogen accumulation in the liver with the aid of Solergy can amplify cellular energy. To assess the impact of Solergy on LMH cells, the glycogen concentration was measured in response to the acute application of glucose and Solergy. The data reveal that short-term application of Solergy induced a less steep rise in the glycogen concentrations compared to glucose, as presented in Fig. 3A. However, the effect of Solergy was more stable and concentration dependent, as demonstrated in Fig. 3B and C. In contrast to glucose, which initially caused more pronounced spikes in glycogen production (acute manner), the effect of Solergy was characterized by a less pronounced increase that persisted over time (Fig. 3D). These findings suggest that Solergy enhances glycogen at the cellular level, and this enhancement was sustained over time in conjunction with increased ATP levels and reduced oxidative stress.

### 3.4 Antioxidant effect

**Fig. 4:**
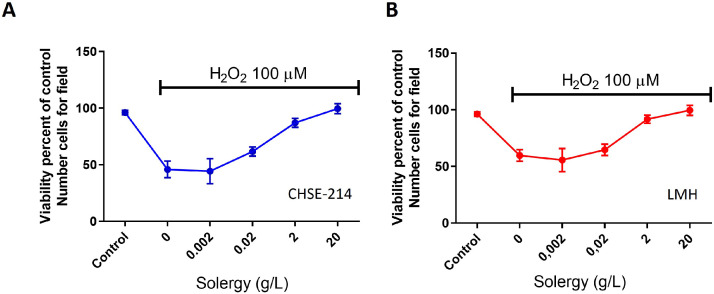
**Antioxidant effect**. Viability of CHSE-214 (A) and LMH (B) cells after 24 h of incubation with H_2_O_2_ in the presence of increasing concentrations of Solergy. The averages ± SEMs are shown.

After recognizing the baseline effect of the product, we evaluated the cellular protection offered by the additive. To this end, CHSE-214 (trout embryo) and LMH (rooster hepatocyte) cell cultures were exposed to a toxic concentration of H_2_O_2_ and increasing concentrations of Solergy. As demonstrated in Fig. 4A and B, cells incubated with Solergy recovered viability, indicating that this additive protected against oxidant-induced damage in both cell lines and suggesting that Solergy protects cells by reducing oxidation.

### 3.5 TBARS values

**Fig. 5:**
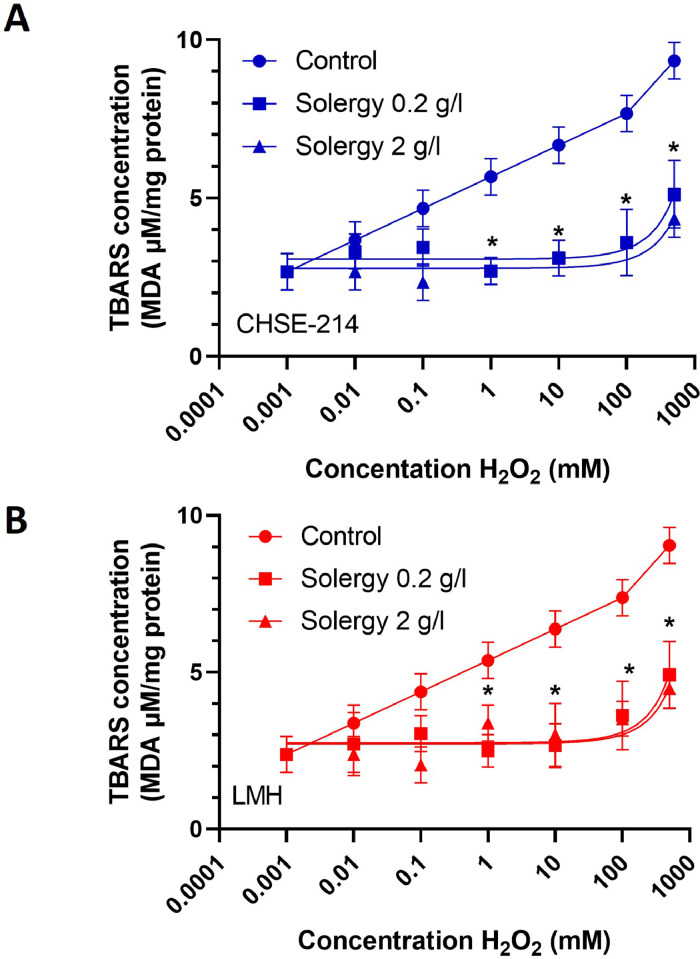
Curves of TBARS values. TBARS concentrations in CHSE-214 (A) and LMH (B) cells incubated with H_2_O_2_ at concentrations ranging from 0.001 to 1000 mM and Solergy at two concentrations. A control is also included for reference. The data points represent the averages ± SEMs.

The reduction in cell mortality after oxidative stress suggests the existence of an antioxidant mechanism of cellular protection that complements the metabolic effect of the product. To assess this behavior of Solergy, TBARS concentration curves were generated for cultures exposed to increasing H_2_O_2_ concentrations in the absence or presence of Solergy at two different concentrations (Fig. 5A and B). Both concentrations of Solergy reduced membrane oxidation and thus cellular aging, implying that this cellular protection mechanism increases cell viability. These findings also suggest that increasing concentrations of Solergy diminish oxidative stress, resulting in reduced lipid oxidation in both cell models.

## 4 Discussion of the results

The results of this study highlight the potential benefits of Solergy, demonstrating its positive impact on cell development through the enhancement of metabolic processes and utilization of ATP for gluconeogenesis regulation. These findings suggest that Solergy could have valuable applications in animal production [1]. The quest for dietary additives with these characteristics has become a priority in the formulation of feed for productive livestock.

The constructed safety curves indicate that the recommended doses of Solergy are not toxic to cells, as illustrated in Fig. 1. This outcome is intriguing because it suggests that Solergy is not associated with baseline toxicity but rather generates a biological response at higher concentrations, which is indicative of its biological activity. Furthermore, the antioxidant effect of Solergy synergizes with its effect on metabolic activation, resulting in improved cell viability. Similar effects have been reported for molecules with comparable mechanisms of action.

This study demonstrates the impact of Solergy on ATP generation in LMH cells, as these additive preserves cell viability even under high metabolic demands (Fig. 2). Solergy enhances ATP production while maintaining the ATP/ADP ratio, indicating the modulation of metabolic activity. As depicted in Fig. 3, Solergy increases the glycogen levels in rooster hepatocytes without compromising cell viability and thereby regulates oxidative stress, and this effect allows the ATP concentration to increase without adversely affecting cell viability or inducing membrane oxidation, as observed for related phenomena [21]. Solergy stimulates metabolic energy production by reducing oxidative stress and maintaining glycogen levels, which indicates the influence of this compound on cellular respiration.

The data suggest that Solergy moderately reduces oxidative stress damage at the recommended doses, which results in decreased mortality associated with oxidative agents such as H_2_O_2_ (Fig. 4). This finding is noteworthy because it shows that Solergy promotes metabolic activity without causing cell death due to the presence of cellular debris. Several studies have suggested an increase in metabolic activity; for example, high glucose generates oxidative stress [22], and glycogen is modulated by glucose levels [23,24]. Although not primarily an antioxidant, Solergy exhibits a protective effect that complements its metabolic impact (Fig. 4). A rise in metabolic activity can lead to an increase in oxidative stress [21], especially during cell division, which is considered a source of oxidative stress [21]. However, Solergy effectively induces increased cell metabolic activity without adverse consequences on viability, which consequently reduces cell mortality caused by oxidative stress and inhibits the oxidative process.

Moreover, as depicted in Fig. 5, the increase in membrane oxidation, as revealed by the TBARS measurements, corresponds proportionally to the increase in the H_2_O_2_ concentration. Nevertheless, in the presence of Solergy, this pattern is not observed with increasing concentrations of H_2_O_2_. Therefore, Solergy exhibits antioxidant properties that mitigate cell oxidation in both cell lines, which enhances their viability.

The data obtained from these cell models collectively indicate that Solergy promotes ATP generation, maintains the ATP/ADP ratio, and elevates the intracellular glycogen concentration. Furthermore, Solergy can reduce oxidative stress damage at the recommended doses and thus functions as an antioxidant that safeguards against cell oxidation and enhances cell viability in both cell lines. This compound has been suggested to be biologically active and safe for use in animals and can thus serve as a viable solution to improve production processes.

In conclusion, these findings support the incorporation of Solergy in feed formulations as a potentially effective strategy to enhance animal production while minimizing cellular oxidation due to oxidative stress through increased metabolic activity. As a next step, a follow-up study will incorporate the additive into animal diets, as previously conducted in other studies, and will use of serum from animals consuming these diets [4,5] to establish a model of the conditioned environment. This study has elucidated the mechanisms of action of Solergy, providing a better understanding of and insights into the use of Solergy in diets and explaining some of the production-level mechanisms already described for similar compounds [6,7]. Although this *in vitro* model cannot address all questions, it serves as a valuable tool for refining the design and determining the practical application of Solergy for *in vivo* experiments.

## CRediT authorship contribution statement

**Pamela Olivares-Ferretti:** Writing – review & editing, Methodology. **Viviana Chavez:** Resources, Supervision, Funding acquisition. **Ekaitz Maguregui:** Project administration, Resources. **Silvia Jiménez:** Project administration, Resources. **Octavi Colom:** Supervision, Validation. **Jorge Parodi:** Writing – original draft, Formal analysis, Conceptualization, Data curation.

## Declaration of Competing Interest

I Jorge Parodi Rivera, the author reports no conflicts of interest. The author alone is responsible for the content and writing of the paper.

## Data Availability

Data will be made available on request.

## Appendix Supplementary materials

Image, application 1
